# Plasma Exchange in ANCA-Associated Vasculitis: A Narrative Review

**DOI:** 10.3390/jcm10215154

**Published:** 2021-11-03

**Authors:** Stathis Tsiakas, Smaragdi Marinaki, Sophia Lionaki, John Boletis

**Affiliations:** 1 Clinic of Nephrology and Renal Transplantation, National and Kapodistrian University of Athens Medical School, Laiko Hospital, 11527 Athens, Greece; smaragdimarinaki@yahoo.com (S.M.); laikneph@laiko.gr (J.B.); 2 2nd Department of Internal Medicine, National and Kapodistrian University of Athens Medical School, Attikon Hospital, 12462 Athens, Greece; sofia.lionaki@gmail.com

**Keywords:** plasma exchange, plasmapheresis, ANCA-associated vasculitis, kidney disease, review

## Abstract

Therapeutic plasma exchange (TPE) is an adjunctive intervention to immunosuppression for the treatment of severe renal involvement or lung hemorrhage in patients with ANCA-associated vasculitis (AAV). Patients with AAV have an increased risk for progression to end-stage kidney disease (ESKD) or death despite advances in immunosuppressive therapy. The potential pathogenicity of ANCA makes TPE a reasonable treatment approach for the life-threatening complications of AAV. The efficacy of intensive TPE in rapidly progressive glomerulonephritis was originally described in small studies almost four decades ago. Further randomized trials examined the addition of TPE to standard of care, exhibiting mixed results in both patient and renal survival. The largest clinical trial to date, PEXIVAS, failed to demonstrate a clear benefit for TPE in severe AAV. In light of new evidence, the role of TPE remains controversial across the vasculitis medical community. The purpose of this review is to summarize the clinical indications and the current available data for the use of TPE in patients with severe AAV.

## 1. Introduction

Therapeutic plasma exchange (TPE) is an extracorporeal procedure in which plasma is separated from other blood constituents and subsequently removed from the patient in replacement of fresh frozen plasma or albumin solutions. The fundamental rationale for TPE lies in the removal of a circulating pathogenic factor, namely an antibody, an immune complex, or a monoclonal protein. This therapeutic potential has led to the use of TPE in a number of autoimmune neurologic, hematologic, and renal disorders [1,2,3,4,5]. In addition to TPE, concomitant immunosuppressive treatment is typically applied in these cases as a means to prevent the production of the culprit immune factor. The level of evidence for therapeutic apheresis varies considerably depending on the disease. Evidence-based clinical guidelines covering common indications, graded by strength of evidence and recommendation, are provided by the American Society for Apheresis (ASFA) guidelines [6].

ANCA-associated vasculitis (AAV) is a systemic disease characterized by destructive inflammation of small- to medium-sized blood vessels in the presence of circulating ANCA [7]. Despite the introduction of immunosuppressive agents since the 1970s, major organ involvement, including kidney and lung, is still related to a significant mortality ratio compared to the general population [8,9]. Given the immunologic nature of AAV, plasma exchange has been applied as an adjunct therapy in patients with active, severe renal disease or diffuse alveolar hemorrhage. A number of clinical studies throughout the years have provided information about the efficacy of TPE in AAV, though with ambiguous outcomes for renal and patient prognosis [10,11,12,13,14,15,16,17,18]. Since TPE is an invasive method of treatment, occasionally complicated by bleeding disorders, infections, hypotension, or anaphylactic reactions, there is an increased need for concrete evidence regarding the use of TPE in AAV [19,20]. 

This narrative review focuses on (i) the pathogenetic rationale, (ii) the current clinical indications, and (iii) the overview of clinical studies of TPE in AAV, as well as (iv) the PEXIVAS trial and (v) its impact on the future of TPE in severe AAV.

## 2. Pathogenetic Rationale for Plasma Exchange in ANCA-Associated Vasculitis

Antineutrophilic cytoplasmic antibodies (ANCA) are mainly immunoglobulin G (IgG) antibodies detected by indirect immunofluorescence or an enzyme-linked immunosorbent assay [21]. The two major antigens targeted by ANCA are proteinase 3 (PR3) and myeloperoxidase (MPO), which are expressed in the neutrophil primary granules and the monocytes. The presumed pathogenetic mechanism of ANCA-induced inflammatory tissue injury includes the binding of ANCA to primed neutrophils, leading to neutrophil degranulation and activation of the alternative complement pathway [22,23]. Several studies in animal models have shown that ANCA apart from potential biomarkers have an actual pathogenetic role in the disease. Specifically, Xiao et al. reported that the infusion of splenocytes from immunized MPO knockout mice or purified anti-MPO IgG antibodies into wild-type mice resulted in the development of severe necrotizing and crescentic glomerulonephritis with a paucity of glomerular immune deposits [24]. In another study, all immunized Wistar Kyoto rats with human MPO developed crescentic glomerulonephritis and lung hemorrhage [25]. Of note, a case of human transplacental transfer of MPO antibodies from a mother to the fetus has been described, causing lung hemorrhage to the neonate [26]. Based on the aforementioned evidence, a direct relationship between ANCA and active disease was suggested.

Considering the pathogenetic essence of ANCA, the removal of these antibodies may exert a beneficial effect on the disease. Immunoglobulin G has a half-life of approximately 21 days; depletion of the serum IgG antibody levels solely by halting their production with immunosuppressive agents would require at least several weeks. TPE offers the possibility of rapidly removing these pathogenic antibodies from the patients’ plasma when added to the standard immunosuppressive regimen. Both common techniques of TPE, centrifugal apheresis and membrane plasma separation, are effective in removing IgG molecules. Due to extravascular distribution, one day intervals between TPE sessions are commonly applied in order to allow immunoglobulins to further redistribute into the vascular space; exceptions to this practice include emergency care circumstances where daily sessions are required, e.g., pulmonary hemorrhage [27,28]. Besides antibody elimination, other putative beneficial mechanisms of action of TPE include the removal of inflammatory mediators, such as cytokines and complement components, and the replenishment of plasma factors via fresh frozen plasma infusion, such as factor H [29].

## 3. Current Clinical Indications for Plasma Exchange in ANCA-Associated Vasculitis

AAV comprises a group of systemic disorders that share a common pathology of pauci-immune necrotizing inflammation of small vessels, resulting in tissue injury and multiple organ involvement. According to the 2012 Revised International Chapel Hill Consensus Conference, AAV is classified into granulomatosis with polyangiitis (GPA), microscopic polyangiitis (MPA), and eosinophilic granulomatosis with polyangiitis (EGPA) [30]. Clinical features may overlap between these entities, especially MPA and GPA. EGPA rarely involves the kidney and is distinguished by the presence of eosinophilia and asthma [31]. 

Kidney disease at diagnosis is common (70% and 90% in GPA and MPA, respectively) and is an important prognostic factor for patient survival. An estimated glomerular filtration rate (eGFR) below 50 mL/min/1.73 m^2^ at presentation correlates with a 50% risk of ESKD or death at 5 years. The typical renal lesion is that of a necrotizing crescentic glomerulonephritis with little or no immune complex deposition, manifesting as a rapid decline in kidney function with proteinuria and active urine sediment. Lungs are also frequently involved, especially in GPA. Diffuse alveolar hemorrhage is a serious complication associated with an increased risk of death, affecting approximately 10% of patients. Before the introduction of immunosuppressive agents, up to 90% of patients died within two years of AAV diagnosis. Cyclophosphamide along with high-dose corticosteroids significantly reduced mortality, although it was associated with serious adverse events, namely infections and malignancy [9,32,33]. 

In the current era, the standard initial immunosuppressive regimen for new-onset AAV consists of cyclophosphamide or rituximab in combination with corticosteroids. The use of TPE is mainly reserved for patients with severe renal impairment or lung hemorrhage in the setting of new or relapsing disease [34]. According to the recently published international *Clinical Practice Guidelines for the Management of Glomerular Diseases* (KDIGO 2021), TPE should be considered in patients presenting with a serum creatinine (SCr) level above 5.7 mg/dL (500 μmol/L), requiring dialysis or with a rapidly increasing SCr, and in those with diffuse alveolar hemorrhage who have hypoxemia [35]. The authors note that no sufficient data exist to support the routine use of TPE in patients with an eGFR < 50 mL/min/1.73m^2^. These clinical practice points were mainly based on the results of the two largest clinical trials addressing the effect of TPE in severe AAV (MEPEX and PEXIVAS) and a meta-analysis of all randomized controlled trials conducted before PEXIVAS publication [14,15,36]. The MEPEX trial results favored the use of TPE in patients presenting with severe renal disease (SCr > 5.7 mg/dL), as did the meta-analysis that showed a reduced incidence of ESKD at 3 and 12 months post-diagnosis with the addition of TPE. On the contrary, the PEXIVAS trial failed to demonstrate a benefit of TPE regarding ESKD or death. Nevertheless, the authors concluded that in disease manifestations associated with high mortality, specifically severe renal disease or lung hemorrhage, treatment with TPE should be considered. On account of the PEXIVAS trial results, ASFA updated its guidelines on the use of TPE in AAV by changing the category recommendation for rapidly progressive glomerulonephritis from I to II, downgrading apheresis as a second-line therapy for this disorder, and also by lowering the grade of evidence from 1A to 1B, i.e., a strong recommendation with moderate quality evidence [37].

Another reported clinical indication for TPE is the overlap syndrome of AAV with anti-GBM antibodies. The coexistence of ANCA and anti-GBM antibodies is common; 5% of ANCA-positive patients also have anti-GBM antibodies, and 32% of anti-GBM-positive patients also have detectable ANCA. Double-positive subjects behave similarly to patients with anti-GBM disease at diagnosis, often presenting with severe acute glomerulonephritis and lung hemorrhage. Nevertheless, they have a tendency to relapse, as seen in AAV patients, and maintenance therapy is advised. The use of TPE should be considered in patients with overlap syndrome, especially those with linear IgG deposition along the glomerular basement membrane in kidney biopsy or diffuse alveolar hemorrhage, until circulating anti-GBM antibodies are no longer detected [38,39,40]. 

Treatment with TPE may play a role in refractory AAV, as defined by the lack of response after at least 4 weeks of standard immunosuppressive regimens and following the exclusion of other factors that may affect treatment response, including nonadherence, infection, and chronic organ damage [40].

## 4. Overview of Clinical Trials of Plasma Exchange in ANCA-Associated Vasculitis 

Plasma exchange has been used for the treatment of AAV for over three decades. Following a small case series about the effect of TPE in rapidly progressive glomerulonephritis in the 1980s [41,42,43], Pusey et al. published in 1991 a randomized controlled trial aiming to determine whether TPE offers an additional benefit to oral immunosuppressives in patients with focal necrotizing glomerulonephritis without anti-GBM antibodies. The study showed that dialysis-dependent individuals (19 out of the 48 recruited subjects) were more likely to recover renal function when treated with TPE, while no difference was observed in non-dialysis patients [10]. In 2001, a randomized trial by Zauner et al. demonstrated that histologic characteristics at diagnosis predicted response to immunosuppressive therapy; however, TPE did not add to the improvement in clinical outcomes [11]. 

One of the largest clinical trials examining the use of TPE for severe AAV was the MEPEX trial published in 2007. The study investigated whether adjunct TPE or pulses of intravenous methylprednisolone were more effective in patients presenting with severe renal involvement (SCr > 5.7 mg/dL). A total of 137 patients with biopsy-proven AAV were randomized to receive seven sessions of TPE versus 3 g of methylprednisolone in addition to oral cyclophosphamide and corticosteroids. The use of TPE was associated with a significant improvement in renal recovery at 3 months (69% in the TPE group VS. 49% in the methylprednisolone group, *p* = 0.02) and a 24% risk reduction of ESKD at 12 months compared to intravenous methylprednisolone. The degree of chronicity at kidney biopsy was predictive of worse renal prognosis. Patient survival was similar, and a high rate of serious adverse events was found in both groups, which was attributed to advanced age, the use of immunosuppressive agents, and the severity of renal impairment. The MEPEX results supported the hypothesis that TPE is more beneficial to patients presenting with severe active kidney disease [15]. Although short-term renal outcomes were quite impressive, a report on extended follow-up of the same trial found that the effect of TPE on kidney disease did not last. After a median follow-up of approximately 4 years, ESKD or death occurred in 68% of the patients, and the incidence did not differ between the groups. Infection was the main cause of mortality, while death from lung hemorrhage was infrequent in both groups. It is to be noted that severe diffuse alveolar hemorrhage was an exclusion criterion of the study [44].

A meta-analysis by Walsh et al. in 2011 examined the role of adjuvant TPE in renal vasculitis, encompassing nine randomized trials, including MEPEX. The pooled relative risk of ESKD or death was 0.80 (95% CI 0.65–0.99; *p* = 0.04) for patients treated with adjunctive TPE compared to standard care alone. The authors underlined a favorable effect of TPE on ESRD but not on mortality, probably explained by a reasonable survival in dialysis or transplantation among AAV patients. However, it was noted that the available data were insufficient to make reliable conclusions as to whether the addition of TPE reduced the composite end point of ESKD or death [45]. 

Further several smaller studies provided useful information regarding the efficacy of TPE in AAV. In 2012, Gregersen et al. demonstrated that early addition of TPE to standard immunosuppressive therapy in PR3-ANCA-positive patients with eGFR < 60 mL/min/1.73 m^2^ improved the composite primary outcome of death, ESKD, and relapses at 1 year [18]. Positive outcomes with the use of TPE as induction therapy were also shown in a randomized trial with 32 GPA patients and less severe renal disease (SCr > 2.85 mg/dL) [17]. A potential benefit of TPE as a rescue therapy in progressive systemic AAV was proposed by de Joode et al. in 2014. In this study of 26 patients, TPE was added to the induction regimen on average 18 days after treatment initiation due to inadequate response, increasing serum creatinine, or progressive pulmonary disease. The introduction of TPE resulted in a rapid decline of ANCA and a significant improvement in renal function (eGFR 44 mL/min/1.73 m^2^ VS 26 mL/min/1.73m2 pre-TPE) without an increased incidence of infections or mortality at 6 months. The effect was sustained with respect to ESKD during long-term follow-up [46]. 

A recently published retrospective cohort study from the Mayo Clinic examined the clinical outcomes of patients with AAV and severe renal involvement (eGFR < 30 mL/min/1.73 m^2^) after initial treatment with cyclophosphamide or rituximab, with or without TPE. A total of 251 patients from 1996 to 2015 with active, biopsy-proven pauci-immune glomerulonephritis were included in the study, 51 of whom were treated with TPE. Patients received one plasma volume plasma exchanges daily for 7 to 14 days. The replacement fluid was albumin, with fresh frozen plasma used for a portion of the replacement according to bleeding risk. None of the patients were treated with a combination of cyclophosphamide and rituximab. Additional TPE to the standard induction regimen did not benefit remission at 6 months or the incidence of ESKD and patient survival at 18 and 24 months [47]. 

## 5. PEXIVAS Trial

Given the lack of available high-quality data regarding the efficacy and safety of adjunct TPE and the increased incidence of ESKD or death despite advances in immunosuppressive therapy, the multicenter randomized PEXIVAS trial was conducted to evaluate the use of TPE in patients who present with severe AAV, as defined by an eGFR < 50 mL/min/1.73m^2^ or diffuse alveolar hemorrhage. The trial had a two-by-two factorial design; randomization included initial treatment with seven sessions of TPE within 14 days or no TPE and a standard-dose glucocorticoid regimen versus a reduced-dose regimen. The primary outcome was death from any cause or ESKD. Patients received either cyclophosphamide or rituximab as induction therapy and one of the two different regimens of oral glucocorticoids. Pulses of intravenous methylprednisolone (1–3 g) were given to all patients. Azathioprine was used as maintenance therapy after induction with cyclophosphamide. Patients in the TPE arm received albumin as a replacement solution, with fresh frozen plasma used only for the final portion of the replacement if bleeding diathesis was present. Additional TPE sessions for ongoing signs and histological evidence of disease activity or serological biomarkers (e.g., elevated ANCA titers) were not permitted.

A total of 704 patients across 16 countries with GPA or MPA (new-onset 91.2% and relapsing 8.9%) and a history of positive ANCA tests (MPO-ANCA 59% and PR3-ANCA 41%) were recruited in the study. Enrollment took place from June 2010 to September 2016. Kidney involvement was almost universal (98%), with one-third of the patients (29%) presenting with severe renal impairment (SCr > 5.7 mg/dL or dialysis dependence). Kidney biopsy was not a prerequisite if the patient had hematuria or proteinuria. The median creatinine level at presentation was 3.27mg/dL. Lung hemorrhage was present in 27% of the patients. Approximately one-third of them had severe pulmonary hemorrhage, as defined by an oxygen saturation ≤ 85% or the need for mechanical ventilation. The presence of anti-GBM antibodies was an exclusion criterion of the study.

Most patients received cyclophosphamide (85%) either intravenously or orally as induction therapy. With regard to TPE, the primary composite endpoint of ESKD or death was met by 28.4% in the TPE group and 31% in the control group (hazard ratio with TPE, 0.86, 95% CI 0.65–1.13, *p* = 0.27) after a median follow-up of 2.9 years. There was no significant difference in the secondary outcomes of ESKD, death from any cause, sustained remission, serious adverse events, and serious infections at 1 year between the two groups. The low-dose glucocorticoid regimen was shown to be non-inferior to the standard regimen concerning the primary endpoint. Significantly fewer serious infections at 1 year were observed in the reduced-dose regimen. 

## 6. The Role of Plasma Exchange in ANCA-Associated Vasculitis after PEXIVAS Trial

The results of the PEXIVAS trial created controversy across the medical community regarding the role of TPE in severe AAV [48,49,50,51,52,53]. The study included the largest patient population to date with a multinational enrollment, which allows for a broad generalizability of the results. However, several limitations of potential clinical significance arose after careful review.

Kidney biopsy was not a requirement at entry, and while most patients had one, analysis of the existing histologic data has not yet been performed to investigate the degree of activity with the response to TPE. Considering, for instance, that patients with MPO-ANCA vasculitis occasionally follow a slowly progressive course and exhibit significant chronic lesions at diagnosis, TPE would not be expected to offer an additional advantage. Another concern is the broad range of renal impairment (eGFR < 50 mL/min/1.73 m^2^) that was allowed in the study. As shown by previous clinical trials, TPE primarily favors those with severe active renal disease at presentation. Subgroup analysis of the primary outcome showed that TPE offered a nominal benefit in patients with a serum creatinine level > 5.7 mg/dL or those requiring dialysis (HR 0.77, CI 0.53–1.11). In view of the marked improvement in the renal outcomes of patients with similar presentation and biopsy-proven AAV shown in the MEPEX trial, TPE may have a favorable effect in subjects who present with severe renal impairment.

Of note, the survival analysis in the PEXIVAS trial showed an advantage, albeit non-significant, of TPE in the first year regarding the primary outcome. Since TPE is a brief intervention of the induction therapy, improvement is anticipated in a short period of time after treatment. Other factors may influence the course of the disease in the long term. With respect to lung hemorrhage, a nominal benefit of TPE was observed in the trial (HR 0.64, CI 0.33–1.24 for nonsevere hemorrhage, HR 0.67, CI 0.28–1.64 for severe hemorrhage). An issue to be considered is if the power of the study was high enough to exhibit a significant effect in subjects with the most severe clinical manifestations. Selection bias may have affected the relatively low recruitment of patients with severe diffuse alveolar hemorrhage in the trial since many physicians would hesitate not to use TPE in this population. Given the low proportion of patients with severe pulmonary hemorrhage and the associated high mortality, TPE may still have a role in the management of this life-threatening complication. 

The PEXIVAS trial undoubtedly enriched the existing knowledge with useful information about the initial management of patients with severe AAV. Despite the reported neutral effect of TPE, other important implications for the attending physicians also came to light. The use of TPE was not associated with more serious adverse events, including infections, as shown by previous studies. Given the potential benefit in selected subgroups with severe renal or pulmonary disease and the lack of undesired effects, TPE will likely continue to be a part of the treatment armamentarium for many specialists. Moreover, the trial demonstrated that the use of a reduced-dose glucocorticoid regimen was safer and equally effective. Lastly, it is plausible that more evidence from PEXIVAS will emerge in the future, as further analysis of the data (e.g., kidney biopsy evaluation) may provide additional insights about the utility of TPE in specific subsets of patients.

## 7. Conclusions

Treatment with TPE along with immunosuppressive drugs comprise the standard induction regimen for patients with AAV who present with rapidly progressive glomerulonephritis or diffuse alveolar hemorrhage. The recent findings of large clinical trials, particularly PEXIVAS, were met with concern among the medical community across the globe, especially nephrologists. In light of the evolving data, the indications of TPE for severe AAV are currently a subject of debate. Novel therapeutic advances in the field, including complement blockade agents, may change the therapeutic strategies in the future [54]. However, until more solid evidence is available from targeted clinical studies, it is our estimation that TPE will continue to be a therapeutic option for subgroups of patients with severe renal disease or pulmonary hemorrhage.

## Data Availability

Not applicable.
